# Pharmacological inhibition of STAT3 pathway ameliorates acute liver injury in vivo via inactivation of inflammatory macrophages and hepatic stellate cells

**DOI:** 10.1096/fba.2019-00070

**Published:** 2020-01-03

**Authors:** Büsra Öztürk Akcora, Alexandros Vassilios Gabriël, Ana Ortiz‐Perez, Ruchi Bansal

**Affiliations:** ^1^ Department of Biomaterials Science and Technology Technical Medical Centre Faculty of Science and Technology University of Twente Enschede The Netherlands; ^2^ Department of Pharmacokinetics, Toxicology and Targeting Groningen Research Institute of Pharmacy University of Groningen Groningen The Netherlands

**Keywords:** acute liver injury, hepatic stellate cells, inflammation, macrophages, STAT3 signaling pathway, WP1066

## Abstract

Liver diseases represent a major health problem worldwide, in particular, acute liver injury is associated with high mortality and morbidity. Inflammatory macrophages and hepatic stellate cells (HSCs) are known to be involved in the pathogenesis of acute liver injury. In this study, we have investigated the implication of STAT3 inhibition in acute liver injury/early fibrogenesis. In fibrotic human livers, we found STAT3 mRNA expression was significantly upregulated and correlated with collagen I expression. In vitro, STAT3 signaling pathway was found to be activated in TGFβ‐activated HSCs and inflammatory macrophages. STAT3 inhibitor, WP1066 significantly inhibited TGFβ‐induced collagen I, vimentin and α‐SMA expression, and contractility in human HSCs. In LPS‐ and IFNγ‐induced pro‐inflammatory macrophages, WP1066 strongly attenuated nitric‐oxide release and expression of major inflammatory markers such as TNF‐α, iNOS, CCL2, IL‐1β, IL‐6, and CCR2. In vivo in CCl_4_‐induced acute liver injury mouse model, WP1066 significantly reduced collagen expression, HSCs activation, and intrahepatic inflammation. Finally, in LPS‐induced human hepatic 3D spheroid model, WP1066 inhibited LPS‐induced fibrotic and inflammatory parameters. In conclusion, our results demonstrate that the therapeutic inhibition of STAT3 pathway using WP1066 targeting HSCs and inflammatory macrophages suggests a potential pharmacological approach for the treatment of acute liver injury.

**Abbreviations**
AEC: 3‐amino‐9‐ethyl carbazole
Arg1: Arginase 1
CCL2/MCP1: chemokine (C‐C motif) ligand 2/Macrophage chemotactic protein‐1
CCl_4_: carbon tetrachloride
CCR2: chemokine (C‐C motif) receptor 2
DAPI: 4',6‐diamidino‐2‐phenylindole
DMSO: Dimethyl sulfoxide
ECM: extracellular matrix
HRP: horseradish peroxidase
HSCs: hepatic stellate cells
IL‐1β: Interleukin 1 beta
IL‐6: Interleukin 6
MHC II: major histocompatibility complex class II
MRC1: mannose receptor 1
mRNA: messenger RNAs
NaCl: sodium chloride
NASH: non‐alcoholic steatohepatitis
NOS2/iNOS: nitric oxide synthase 2
qRT‐PCR: quantitative real‐time polymerase chain reaction
SDS: sodium dodecyl sulfate
TGFβ: transforming growth factor beta
Tris‐HCl: Tris(hydroxymethyl)aminomethane hydrochloride
YM1: Beta‐N‐acetyl hexosaminidase or Chi3l3, Chitinase‐3‐like protein 3
α‐SMA: alpha smooth muscle actin

## 1 INTRODUCTION

Acute liver injury represents a major health problem worldwide with substantial morbidity and mortality.^1^, ^2^ It is characterized by an excessive accumulation of extracellular matrix (ECM), activation of hepatic stellate cells (HSCs), inflammatory cell infiltration, and transformation of hepatic architecture. During acute liver injury, these changes are transient, and the process is reversible. However, upon chronic injury, the progressive substitution of parenchyma by scar tissue leads to cirrhosis and end‐stage liver failure that has poor outcome and usually requires liver transplantation. During early fibrogenesis, HSCs become activated and differentiate into proliferative and contractile myofibroblasts that are the main drivers of fibrosis.^3^, ^4^

Besides HSCs, hepatic macrophages are the core mediators in the pathogenesis of acute or chronic liver injury.^5^, ^6^, ^7^ Macrophage infiltration, function, and differentiation are a dynamic process; macrophages respond to the environmental signals from tissue in a diversified manner giving rise to different polarization states.^5^, ^6^, ^7^ Pro‐inflammatory M1 macrophages have been recognized in the progression of acute liver injury, and can rapidly polarize into restorative M2 macrophages upon termination of liver injury that stimulate tissue repair.^5^, ^8^

Although the molecular mechanisms of fibroblast activation are not completely understood, transforming growth factor‐β (TGFβ) is recognized as a core promotor of fibrosis.^9^ Stimulation with TGFβ induces fibroblasts activation and differentiation to myofibroblast phenotype with distinct gene expression profile.^9^ The signal transducer and activator of transcription 3 (STAT3) signaling pathway integrates several profibrotic signals and has been identified as a key mediator of fibrosis.^10^ STAT3 signaling pathway has been shown to be activated by multiple factors that are crucial for liver disease such as interleukin‐6 (IL‐6), interleukin‐10, and growth factors including TGFβ.^10^, ^11^, ^12^ STAT3 signaling pathway has shown to regulate the number of fundamental cellular processes for example, inflammation, growth and apoptosis, proliferation, differentiation, and migration. Importantly, the involvement of STAT3 pathway in various diseases has encouraged the development of various STAT3 antagonists, several of which are highly promising for therapeutic intervention.^11^, ^13^, ^14^

In this study, we have demonstrated that upregulation of STAT3 in liver fibrosis patients, in TGFβ‐activated HSCs and LPS‐ and IFNγ‐stimulated proinflammatory M1 macrophages*,* and in CCl_4_‐induced liver injury mouse model. Pharmacological inhibition of STAT3 signaling pathway with WP1066, a selective STAT3 antagonist, significantly inhibited inflammatory macrophages and TGFβ‐induced HSCs activation in vitro*,* and attenuated early fibrogenesis and inflammation in acute CCl_4_‐induced liver injury mouse model in vivo. Furthermore, WP1066 ameliorated fibrogenesis and inflammatory markers in LPS‐induced human hepatic 3D‐spheroid model.

## 2 MATERIALS AND METHODS

### 2.1 Cell lines

Human hepatic stellate cells (LX2 cells) provided by Prof. Scott Friedman (Mount Sinai Hospital) were cultured in DMEM‐Glutamax medium (Invitrogen) supplemented with 10% fetal bovine serum (FBS, Lonza), and antibiotics (50 U/mL Penicillin and 50 µg/mL streptomycin, Sigma). Murine NIH3T3 fibroblasts and murine RAW264.7 macrophages were obtained from American Type Culture Collection (ATCC). The 3T3 cells and RAW cells were cultured in Dulbecco's modified Eagle's (DMEM) medium (Lonza) and Roswell Park Memorial Institute (RPMI) 1640 medium (Lonza) respectively and supplemented with 2 mmol/L L‐glutamine (Sigma), 10% FBS (Lonza) and antibiotics (50 U/mL Penicillin and 50 µg/mL streptomycin, Sigma).

### 2.2 Effects of STAT3 inhibitor WP1066 on mouse 3T3 fibroblasts and human LX2 cells

The STAT3 inhibitor WP1066 used in this study was purchased from Selleckchem. Cells were seeded in 24‐well plates (5 × 10^4^ cells/well) and 12‐well plates (1 × 10^5^ cells/well) and cultured overnight. To assess the effects of the inhibitor, cells were starved overnight with serum‐free medium and then incubated with starvation medium alone, 5 ng/mL of human recombinant TGFβ1 (Roche) with and without 5 µmol/L and 10 µmol/L WP1066 for 24 hours. Cells (24‐well plates) were then fixed with chilled acetone: methanol (1:1), dried and stained for different markers (collagen‐I, α‐SMA, and vimentin) (antibodies are summarized in Table S1). In addition, cells (12‐well plates) were lysed with RNA lysis buffer to perform quantitative real‐time PCR analyses or protein lysis buffer for western blot analyses.

### 2.3 3D collagen‐I gel contraction assay

Collagen‐I suspension (5.0 mL) containing 3.0 mL Collagen G1 (5 mg/mL, Matrix biosciences), 0.5 mL 10× M199 medium, 85 µL 1N NaOH (Sigma), and sterile water was prepared, and then mixed with 1.0 mL (2 × 10^6^) LX2 cells. Collagen gel cell suspension (0.6 mL/well) was added a 24‐well culture plate and allowed to polymerize for 1 hour at 37°C. Polymerized gel was then incubated with 1 mL of serum‐free medium with or without TGFβ (5 ng/mL) together with 10 µmol/L WP1066 followed by detachment of the gels from the culture wells. Photographs were taken using a digital camera at 72 hours. The size of the gels was digitally measured and normalized with their respective well size in each image.

### 2.4 Effects of STAT3 inhibitor WP1066 on differentiated RAW macrophages

RAW macrophages were plated in 12 well plates (1 × 10^5^ cells/well) and cultured overnight at 37°C/5% CO_2_. To assess the effects of the inhibitor, cells were incubated with medium alone, M1, or inflammatory stimulus (10 ng/mL of mouse IFNγ and 10 ng/mL LPS) with and/or without WP1066 (0.5, 1.0, 5.0, and 10.0 μmol/L) for 24 hours. Cells were lysed with RNA lysis buffer to perform quantitative real‐time PCR analyses or with protein lysis buffer for western blot analyses.

### 2.5 Cytokine detection

Measurement of TNF‐α and IL‐6 in macrophage conditioned medium was performed using ELISA kits according to the manufacturer's instructions (Invitrogen). Briefly, RAW macrophages were incubated with medium alone, M2 or restorative stimulus (10 ng/mL of murine IL‐4 and 10 ng/mL IL‐13), and M1 or inflammatory stimulus (10 ng/mL of murine IFNγ and 10 ng/mL LPS) with and/or without WP1066 (5.0 μmol/L) for 24 hours. Conditioned medium/culture supernatant was collected and stored at −80°C until use. This ELISA assay uses the quantitative sandwich immunoassay technique. By comparing the absorbance of the samples to the standard curve, the concentration of the cytokines in culture supernatant was determined.

### 2.6 Effects of STAT3 inhibitor WP1066 on Nitric Oxide (NO) release

The effect of WP1066 on M1 inflammatory macrophages was assessed by measuring the inhibition in nitrite NO_2_ release, a stable NO metabolite produced by M1 inflammatory macrophages. RAW cells (2 × 10^5^ cells/200 μL/well) seeded in 96‐well plates were cultured with IFNγ (10 ng/mL) and LPS (10 ng/mL) together with different concentrations of STAT3 inhibitor WP1066 (0, 0.5, 1, 5, and 10 μmol/L). After 24 hours, 100 μL of culture supernatant was added to 100 μL of Griess reagent (1% sulfanilamide; 0.1% naphthylethylendiamine dihydrochloride; 3% phosphoric acid) and absorbance at 540 nm was measured using microplate reader.

#### 2.6.1 Cell viability assay in human LX2 cells and mouse macrophages

To assess the effects of the inhibitor on cell viability, cells were plated in 96‐well plates and incubated with different concentrations of WP1066 (0.1, 0.5, 1.0, 2.5, 5, 10, and 25 μmol/L) for 24 hours. Cell viability assay was performed using Alamar Blue reagent (Invitrogen) as per manufacturer's instructions. The results are represented as % cell viability normalized to control cells (at 100%) as indicated in the figure legends.

### 2.7 Animals and ethical approval

About 6‐ to 8‐week‐old C57BL/6 male mice weighing 18‐20 g were obtained from Harlan. All the animal experiments in this study were performed in strict accordance with the guidelines and regulations for the Care and Use of Laboratory Animals, Utrecht University, The Netherlands and comply with the guidelines of ARRIVE and the National Institutes of Health. The animal experimental protocols were approved by the Institutional Animal Ethics Committee of the University of Twente, the Netherlands. Animals were housed in a standard animal housing facility under the conditions of constant temperature of (21 ± 2°C) and humidity (60 ± 5), a 12 h light/12 h dark cycles with ad libitum normal diet. Animals were allowed to acclimatize for 2 weeks before the experiment.

### 2.8 CCl_4_‐induced acute liver injury mouse model

To study the effect of WP1066, male C57BL/6 mice were treated with a single intraperitoneal injection of olive oil or CCl_4_ (1 mL/kg in olive‐oil) at day 1. At day 2 and day 3, CCl_4_‐treated mice received intraperitoneal administration of 5 mg/kg WP1066 prepared in 1% DMSO (Sigma) and 5% β‐hydroxy cyclodextrin (Sigma) or vehicle treatment (1%DMSO/5% β‐hydroxy cyclodextrin/PBS) (n = 5 per group). At day 4, all mice were sacrificed, and livers were harvested for the subsequent analysis.

#### 2.8.1 In vitro human 3D spheroid model

To prepare the human 3D spheroids, human hepatocytes HepG2 (80%), human monocytes THP1 (10%), human hepatic stellate cells LX2 (5%), and human umbilical vein endothelial cells HUVECs (5%) were mixed in complete medium as described elsewhere.^15^ Spheroids with a total of 1200 cells were plated in ultra‐low adhesion U bottomed plates (ULA) (1200 cells/100 μL) followed by addition of 100 μL of complete medium. After 7 days of culturing, 3D spheroids were incubated with 10 μg/mL LPS with and without WP1066 (10, 25 and 50 μmol/L). After 7 days of culturing, the spheroids were embedded in Tissue‐Tek optimum‐cutting temperature (OCT) medium and snap frozen in 2‐methyl butane chilled on a dry ice. The spheroids were cryosectioned into 5 μm cryosections and stained. The 3D human spheroid model is schematically presented in Figure 7A.

### 2.9 Immunohistochemistry

Liver tissues were harvested and transferred to Tissue‐Tek optimum‐cutting temperature (OCT) embedding medium (Sakura Finetek), and snap‐frozen in 2‐methyl butane chilled on a dry ice. Cryosections (5 μm) were cut using a Leica CM 3050 cryostat (Leica Microsystems). The sections were air‐dried and fixed with acetone for 20 minutes. Cells or tissue sections were rehydrated with PBS and incubated with the primary antibody (refer to Table S1) overnight at 4°C. Cells or sections were then incubated with horseradish peroxidase (HRP)‐conjugated secondary antibody for 1 hour at room temperature and incubated with HRP‐conjugated tertiary antibody for 1 hour at room temperature. Thereafter, peroxidase activity was developed using AEC (3‐amino‐9‐ethyl carbazole) substrate kit (Life Technologies) for 20 minutes and nuclei were counterstained with hematoxylin (Sigma). For liver sections, endogenous peroxidase activity was blocked by 3% H_2_O_2_ prepared in methanol. Cells or sections were mounted with Aquatex mounting medium (Merck). The staining was visualized, and the images were captured using light microscopy (Nikon eclipse E600 microscope, Nikon). Furthermore, sections were scanned using Hamamatsu NanoZoomer Digital slide scanner 2.0HT (Hamamatsu Photonics, Bridgewater) for quantitative histological analysis. High‐resolution scans were viewed using NanoZoomer Digital Pathology (NDP2.0) viewer software (Hamamatsu Photonics). About 20 images (100×) of each entire section (from NDP) were imported into ImageJ and were analyzed quantitatively at a fixed threshold. All the primary antibodies used in this study have been pre‐tested for specificity. The stainings performed in the study included the negative control (without primary antibody) to confirm the specificity of the staining and showed no non‐specific staining.

### 2.10 Western blot analysis

Cells were lysed using standard western blot lysis buffer (Life Technologies) and the prepared cell lysates were stored at −80°C until use. The samples were boiled and subjected to SDS‐PAGE with 10% Tris‐glycine gels (Life Technologies) followed by protein transfer onto polyvinylidene difluoride (PVDF) membrane. The membranes were developed according to the standard protocols using primary and secondary antibodies as mentioned in Table S2. The bands were visualized using enhanced chemiluminescence (ECL) detection reagent (Perkin Elmer Inc) and photographed using FluorChem M Imaging System (ProteinSimple, Alpha Innotech). Intensity of individual bands was quantified using ImageJ densitometry software and expressed in %.

### 2.11 RNA extraction, reverse transcription, and quantitative real time PCR

Total RNA from cells and liver tissues was isolated using GenElute Total RNA Miniprep Kit (Sigma) and SV total RNA isolation system (Promega Corporation) respectively according to manufacturer's instructions. The RNA concentration was quantitated by a UV spectrophotometer (NanoDrop Technologies). Total RNA (1 μg) was reverse transcribed using iScript cDNA Synthesis Kit (Bio‐Rad). All the primers were purchased from Sigma‐Genosys. Real‐time PCR was performed using 2× SensiMix SYBR and Fluorescein Kit (Bioline, QT615‐05), 20 ng cDNA and pre‐tested gene‐specific primer sets (Tables S3 and S4). The cycling conditions for the BioRad CFX384 Real‐Time PCR detection system were 95°C for 10 minutes, 40 cycles of 95°C/15 sec, 58°C/15 sec, and 72°C/15 sec. Finally, cycle threshold (Ct) values were normalized to reference gene GAPDH and fold changes in expression were calculated using the 2^−ΔΔCt^ method.

### 2.12 Transcriptomic mRNA expression analysis in the human liver tissues

STAT3, collagen‐I, and α‐SMA (*ACTA2*) mRNA expression were assessed in the publicly available transcriptome datasets of liver tissue from patients with fibrosis (GSE14323)^16^ obtained from the National Center for Biotechnology Information Gene Expression Omnibus database (http://www.ncbi.nlm.nih.gov/geo). Patients were categorized into two groups: normal (n = 8) and cirrhosis (n = 13), and data were analyzed using online database GEO2R.

### 2.13 Statistical analyses

All the results are presented as the mean + standard error of the mean (SEM). The graphs and statistical analyses were performed using GraphPad Prism version 5.02 (GraphPad Prism Software). The comparison to the control group was analyzed using unpaired Student's *t* test while multiple comparisons between different groups were performed by one‐way analysis of variance (ANOVA) with Bonferroni post hoc test. The differences were considered significant at *P* < .05. Correlations were assessed using non‐parametric spearman's correlative analysis with Gaussian approximation. The dot plots for the correlation were generated using the GraphPad Prism and represented with Spearman *r* and two‐tailed *P* value. For in vitro experiments, no statistical method was used to predetermine the sample size. For in vivo experiments, the sample size was estimated using power analysis—that is, a difference of 20% with a power of 80% (1 − *β*) and an *α* of .05. For all in vivo studies, mice were blindly randomized into different groups. The investigators were not blinded to allocation for the in vivo experiments but were blinded to allocation for immunohistochemical analyses. Quantitative data analysis was performed in a blinded manner. No samples that were fully processed for different assays, western blotting, or immunohistochemical analysis were excluded. No mice that completed the studies were excluded from the analyses. The data and statistical analysis comply with the recommendations on experimental design and analysis in pharmacology.^17^, ^18^

## 3 RESULTS

### 3.1 Upregulation of STAT3 in human fibrotic patients

**Figure 1:**
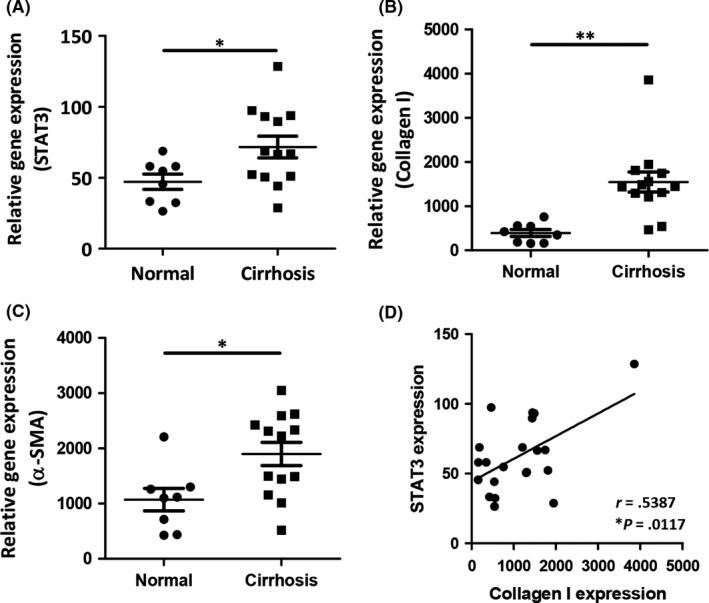
Upregulation of STAT3 expression in cirrhosis patient liver. Relative gene expression of (A) STAT3, (B) COL1A1 (collagen type I alpha 1), (C) *ACTA2* (alpha smooth muscle actin), and (D) correlative analysis of STAT3 and collagen‐I expression of cirrhotic liver tissues vs healthy controls. Data are presented as mean ± SEM; control (n = 8) and cirrhosis (n = 13). *P < .05, **P < .01 denotes significance. *r* denotes Spearman's coefficient and *P* denotes two‐tailed *P* value calculated using non‐parametric Spearman's correlative analysis with Gaussian approximation

We have first assessed the expression of STAT3 in human cirrhotic livers using transcriptomic analysis of the publicly available transcriptome datasets (GSE14323).^16^ We found significantly increased mRNA expression levels of STAT3 (Figure 1A) in human cirrhotic livers as compared to normal livers consistent with induced collagen I and α‐SMA, alpha smooth muscle actin gene *ACTA2* (alpha‐actin‐2) expression (Figure 1B‐C). Overexpression of collagen I, a major ECM protein, is characteristic of liver cirrhosis and α‐SMA, a HSCs activation marker, signifies stellate cell activation and has been described as a marker to determine the degree of liver damage.^19^, ^20^ We further performed a correlative analysis between STAT3 and collagen‐I expression which showed a direct correlation between STAT3 expression and cirrhosis (Figure 1D). These findings indicate a strong association between the STAT3 signaling pathway and liver cirrhosis. However, no significant correlation between STAT3 and α‐SMA expression was observed suggesting that STAT3 expression is modulated by other contributing factors/cells and is not directly regulated by HSCs (Figure S1).

### 3.2 STAT3 signaling pathway inhibitor WP1066 attenuated TGFβ‐induced mouse 3T3 fibroblast activation

**Figure 2:**
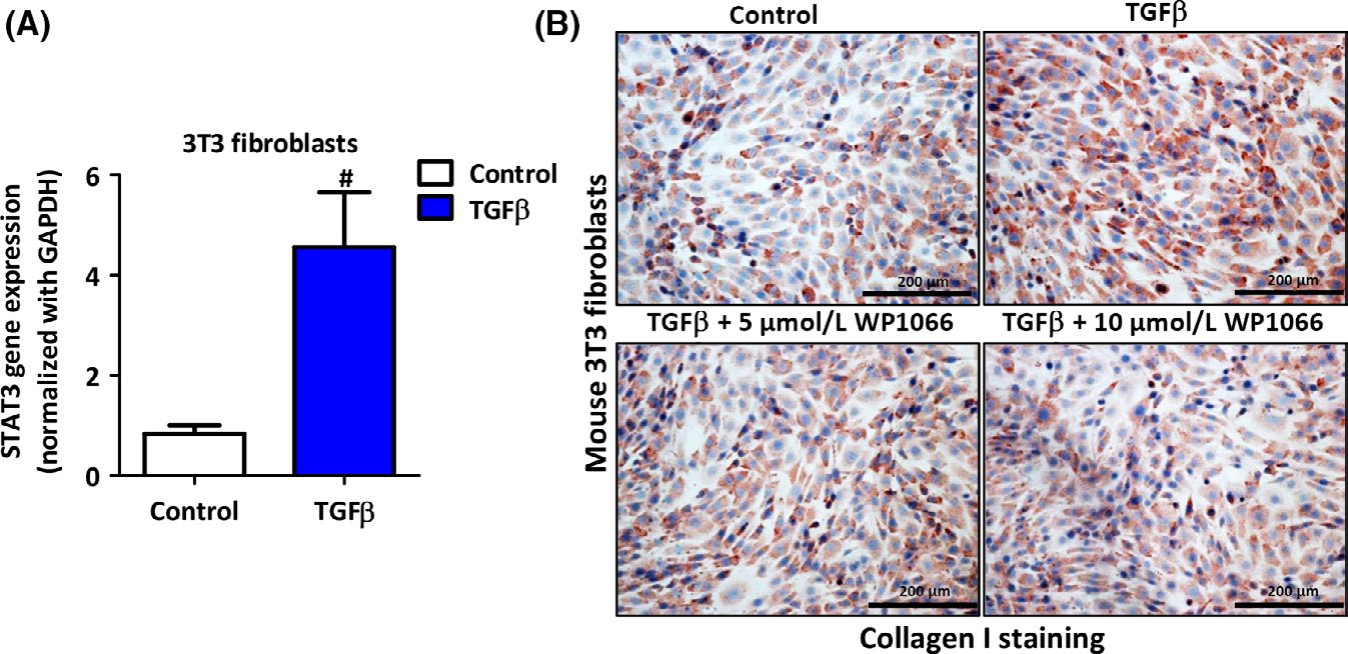
Inhibitory effect of WP1066 on TGFβ activated 3T3 fibroblasts. (A) Relative gene expression of STAT3 in mouse 3T3 fibroblasts with and without TGFβ activation. Data are presented as the mean ± SEM (n = 5). ^#^ *P* < .05 denotes significance vs control. (B) Representative images (n = 5) of Collagen I protein staining in control and TFGβ (5ng/mL) activated murine 3T3 fibroblasts with and without treatment with STAT3 inhibitor, WP1066 (5 and 10 µmol/L)

Transforming growth factor beta 1 (TGF‐β1) is a potent mediator of tissue repair and wound healing by activating fibroblasts to stimulate synthesis of extracellular components and has been implicated in liver diseases.^21^, ^22^ Therefore, we used TGFβ to activate fibroblasts. We examined STAT3 gene expression in TGFβ‐activated 3T3 fibroblasts and found highly increased levels of STAT3 gene expression as compared to control 3T3 fibroblasts (Figure 2A). Thereafter, we investigated the effect of a selective small molecule STAT3 inhibitor, WP1066, on activated mouse 3T3 fibroblasts. Following TGFβ activation, we observed highly significant upregulation of collagen I as compared to control (Figure 2B). Subsequently, treatment with WP1066 dose dependently diminished TGFβ‐induced collagen I expression (Figure 2B; Figure S2). These findings suggest an important role of STAT3 pathway in TGFβ‐induced fibroblast activation.

### 3.3 WP1066 inhibition of human HSCs activation and contractility in vitro

**Figure 3:**
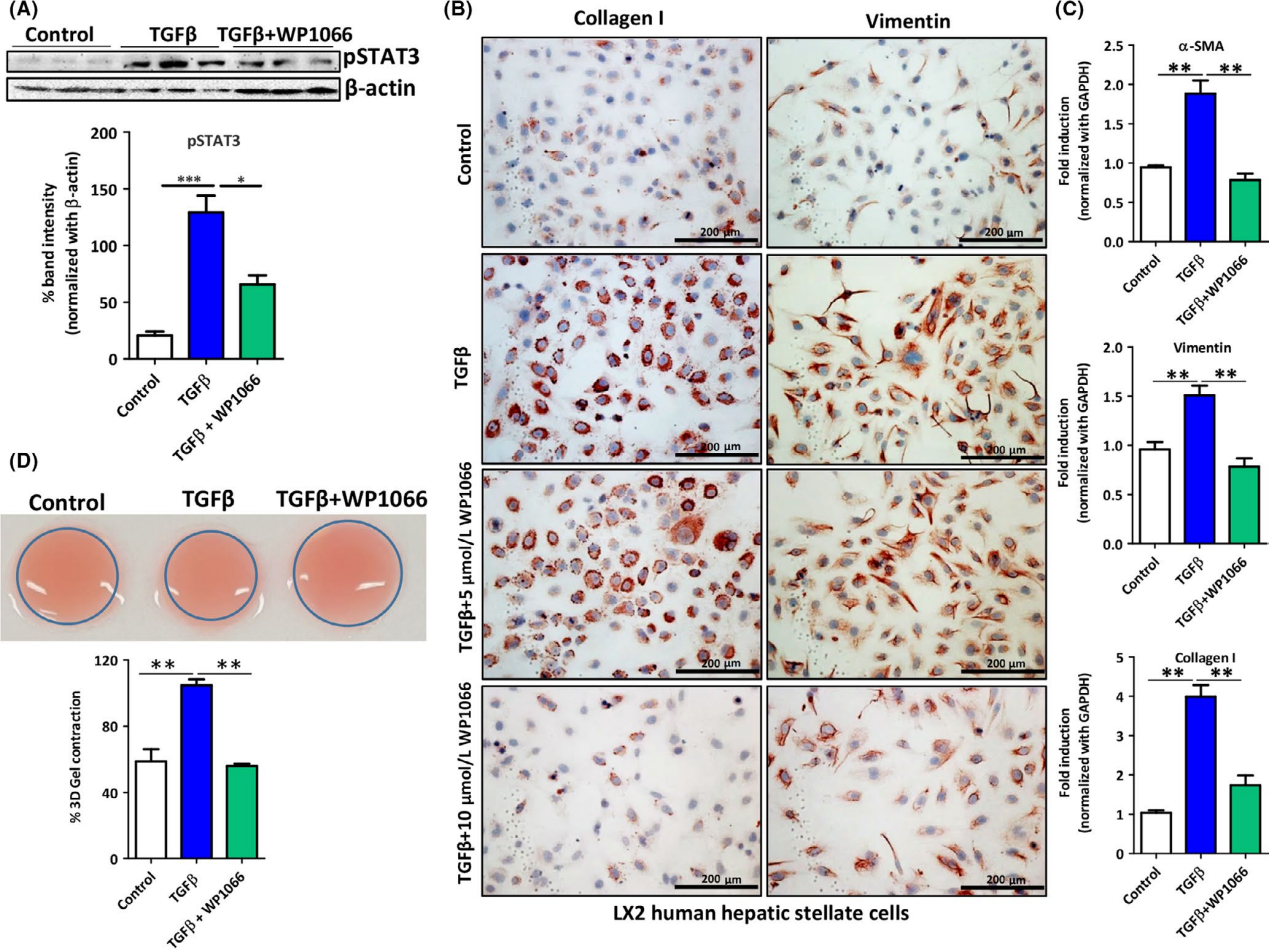
WP1066 inhibits TGFβ‐induced activation and contractility of human HSC (LX2) cells. (A) Figure depicting the western blot bands and quantitative protein expression of phosphorylated STAT3 and β‐actin (as protein normalization control) as analyzed in LX2 cells treated with medium alone (control), TGFβ with and without 10 μmol/L WP1066. Data are presented as the mean ± SEM (n = 5). *P < .05, ***P < .001 denotes significance. (B) Representative images (n = 5) of collagen I and vimentin stained control and TFGβ (5 ng/mL) activated LX2 cells with and without treatment with STAT3 inhibitor WP1066 (5 and 10 µmom/L). (C) Relative mRNA expression for α‐SMA, vimentin, and collagen I in control and TFGβ (5 ng/mL) activated LX2 cells with and without treatment with 10 µmol/L STAT3 inhibitor WP1066. Data are presented as the mean ± SEM (n = 5). **P < .05 denotes significance. (D) Representative images and quantitative analysis of 3D collagen‐I gel contraction of LX2 cells after 72 h following different treatments as indicated. Data are presented as the mean ± SEM (n = 5). **P < .01 denotes significance

We then investigated the activation of the STAT3 signaling pathway in immortalized human hepatic (LX2) stellate cells. We observed that following TGFβ activation, LX2 cells significantly increased STAT3 phosphorylation suggesting the TGFβ‐induced STAT3 pathway activation (Figure 3A). As expected, TGFβ‐induced STAT3 phosphorylation was significantly inhibited following treatment with 10 μmol/L WP1066 (Figure 3A). We further evaluated the effect of STAT3 inhibition in human LX2 cells. Following TGFβ‐induced LX2 activation, we observed highly significant increase in protein expression of collagen I and vimentin, which was dose dependently inhibited after treatment with WP1066 (Figure 3B; Figure S3). mRNA analysis for collagen I, vimentin, and α‐SMA reflected the protein expression, whereby treatment with TGFβ triggered increased mRNA expression for collagen‐I, vimentin, and α‐SMA which was significantly attenuated after treatment with 10 μmol/L WP1066 (Figure 3C). Following injury, HSC transdifferentiate into contractile myofibroblasts that aggravate liver stiffness.^23^ We, therefore, also investigated the effect of STAT3 inhibition on the contractility of HSCs using the 3D‐collagen gel contraction assay. Remarkably, we found that TGFβ‐induced HSCs contractility was significantly attenuated following STAT3 inhibition using WP1066 as analyzed after 72 hours (Figure 3D). These results further confirm the significance of STAT3 signaling pathway in TGFβ‐induced activation of human HSCs. Notably, WP1066 did not induce any significant change in the cell viability at 0‐10 μmol/L doses, however 25 μmol/L WP1066 reduced the cell viability significantly (Figure S4).

### 3.4 STAT3 signaling pathway inhibits LPS‐ and IFNγ‐induced M1 macrophage polarization

**Figure 4:**
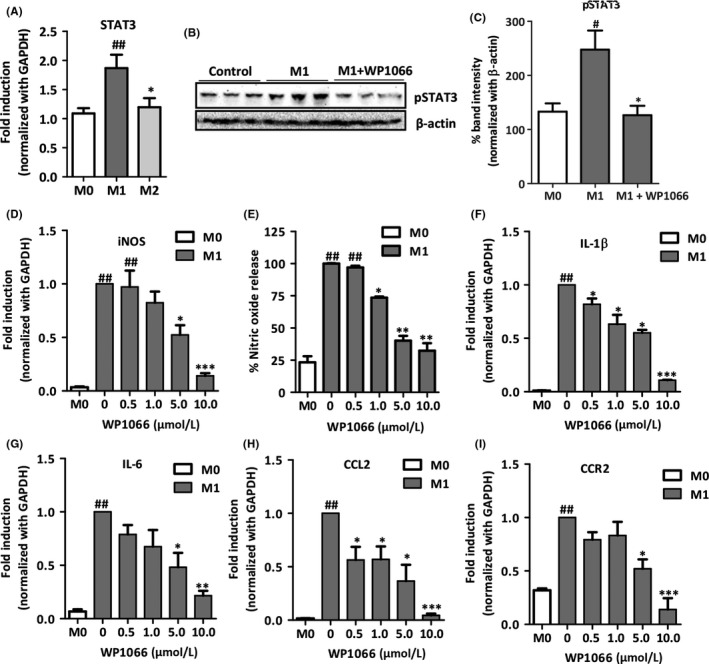
STAT3 inhibitor WP1066 attenuates gene and protein expression of inflammatory markers and nitric oxide release in LPS‐ and IFNγ‐stimulated mouse RAW macrophages. (A) Relative gene expression of STAT3 in M0 (control), M1 inflammatory (LPS and IFNγ treated), and M2 restorative (IL‐4 and IL‐13 treated) macrophages. Data are presented as the mean ± SEM (n = 5). ^##^ *P* < .01 denotes significance vs M0 (control macrophages) and *P < .05 denotes significance vs M1 inflammatory macrophages. Western blot bands (B) and quantitative protein expression (C) of phosphorylated STAT3 and β‐actin as analyzed in M0 and M1 macrophages treated with and without 10 μmol/L WP1066. Data are presented as the mean ± SEM (n = 5). ^#^ *P* < .05 denotes significance vs M0 (control macrophages) and *P < .05 denotes significance vs M1 macrophages. (D) Nitric oxide release analyzed in supernatant of RAW cells that were incubated with medium alone, M1 stimulus (10 ng/mL LPS and 10 ng/mL IFNγ) without and with WP1066 (0, 0.5, 1, 1.5, 5, and 10 μmol/L). Data are presented as the mean ± SEM (n = 5). ^##^ *P* < .01 denotes significance vs M0 (control macrophages) and *P < .05, **P < .01 denotes significance vs M1 macrophages. Relative gene expression in M0, M1, and M1 treated with WP1066 (0, 0.5, 1, 1.5, 5, and 10 μmol/L) for iNOS (E), IL‐1β (F), CCL2 (G), (CCR2) (H), and IL‐6 (I). Data are presented as the mean ± SEM (n = 5). ^##^ *P* < .01 denotes significance versus M0 (control macrophages) and *P < .05, **P < .01, ***P < .001 denotes significance vs M1 inflammatory macrophages

We then examined the role of STAT3 signaling pathway in differentially polarized macrophages. Initially, we investigated the expression of STAT3 in unpolarized M0 macrophages, LPS/IFNγ‐induced M1 inflammatory macrophages, and IL4/IL13‐induced restorative M2 macrophages. We observed increased levels of STAT3 expression in M1 inflammatory macrophages, indicating that STAT3 is associated with inflammation (Figure 4A). Moreover, we investigated the activation of STAT3 pathway (STAT3 phosphorylation) in M1 macrophages and found that LPS/IFNγ‐induced STAT3 phosphorylation which was significantly attenuated by WP1066 (Figure 4B,C). We then investigated the implication of STAT3 inhibition in inflammatory macrophages. We found that WP1066 dose dependently inhibited M1 polarization as illustrated in M1‐specific pro‐inflammatory “signature” markers: nitric oxide (NO) release, mRNA expression of inducible nitric oxide synthase (iNOS), Interleukin 1 beta (IL‐1β), C‐C motif chemokine ligand 2 (CCL2), C‐C chemokine receptor type 2 (CCR2), and Interleukin 6 (IL‐6) (Figure 4D‐I). Subsequently, we investigated secretion of pro‐inflammatory cytokines TNF‐alpha and IL‐6 by M1 polarized macrophages. After treatment with STAT 3 inhibitor WP1066, we observed a significant reduction in both TNF‐alpha and IL‐6 levels (Figure S5). We also assessed the effect of WP1066 on the cell viability of RAW macrophages at different concentrations. We found that drug concentrations of 0‐5 μmol/L showed no significant effects on the cell viability of macrophages, however, at 10 μmol/L dose, cell viability was reduced to approximately 75% compared to M1 macrophages. This reduction in cell viability might be due to the reduced metabolic activity, however, is still much higher than M0 macrophages. Notably, 25 μmol/L and 50 μmol/L doses, showed highly significant reduction in cell viability (Figure S6) indicating toxic effects of WP1066 at higher doses.

### 3.5 In vivo STAT3 signaling pathway inhibition alleviates fibrogenesis in acute liver injury mouse model

**Figure 5:**
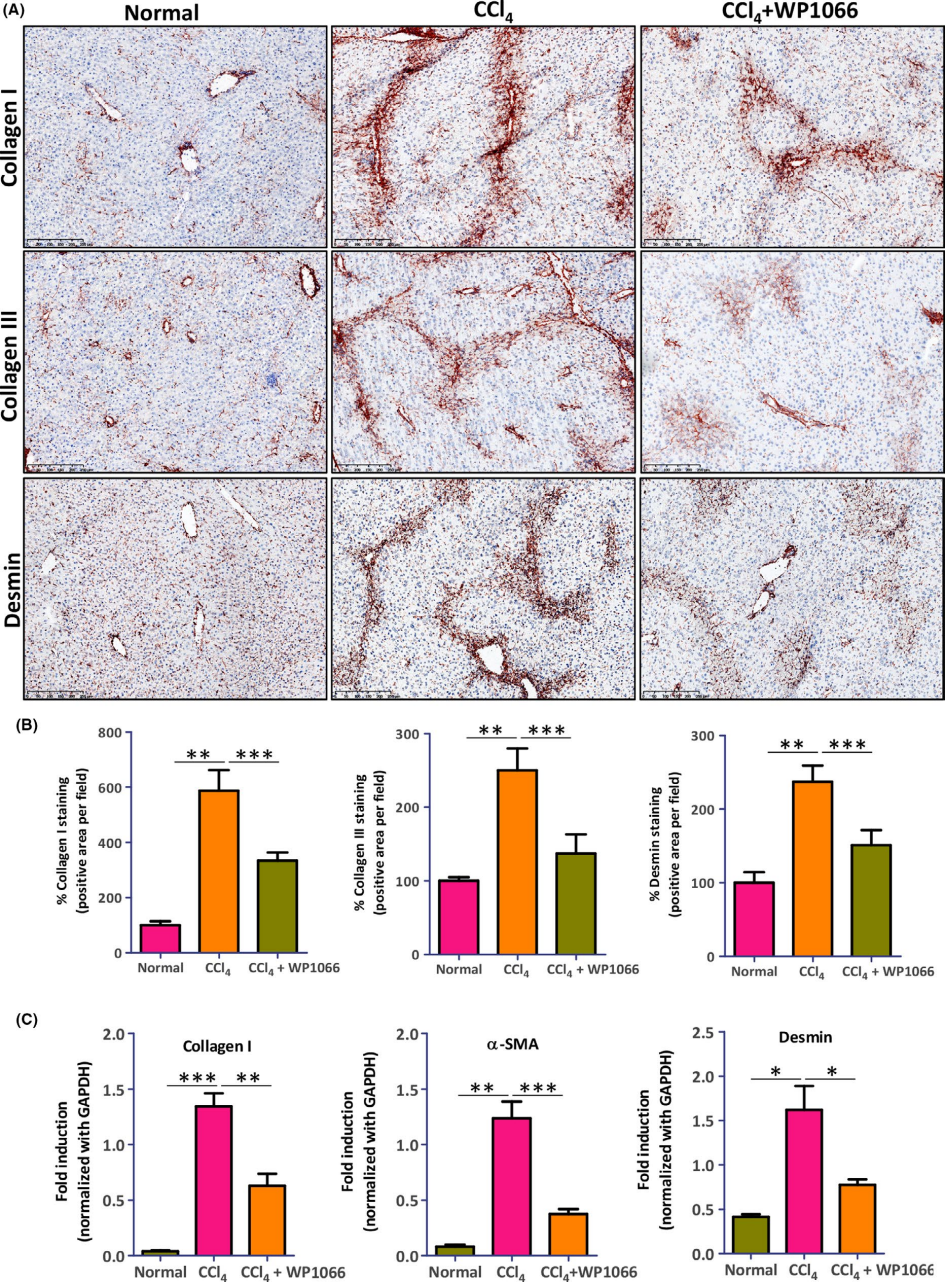
STAT3 antagonist WP1066 inhibits fibrogenesis in acute CCl_4_‐induced liver fibrogenesis mouse model. (A) Representative microscopic images (n = 5 per group) and (B) quantitative analysis of collagen I, collagen III, and desmin stained liver sections from normal and CCl_4_ mice with and without WP1066 treatment. Data are presented as the mean ± SEM (n = 5 per group). **P < .01, ***P < .001 denotes significance. (C) Relative gene expression for collagen‐I, α‐SMA (ACTA2) and desmin in normal and CCl_4_ mice with and without WP1066 treatment. Data are presented as the mean ± SEM (n = 5 per group). *P < .05, **P < .01, ***P < .001 denotes significance

To confirm the above described findings in vivo, we investigated the effects of WP1066 in acute liver injury mouse model. Carbon tetrachloride (CCl_4_) injection induced spontaneous liver damage with acute HSC activation thereby resulting in the elevated levels of intrahepatic protein expression of collagen I, collagen III, and desmin compared to the olive oil administered control livers, as evident by the staining and the respective quantitative analysis (Figure 5A,B). Notably, in vivo treatment with WP1066 (5 mg/kg) significantly reduced collagen I, collagen III, and desmin expression (Figure 5A,B). The protein expression levels were further confirmed by analyzing intra‐hepatic mRNA expression for collagen I, α‐SMA (*ACTA2* gene), and desmin, and that treatment with WP1066 inhibited CCl_4_‐induced gene expression of major fibrotic markers (Figure 5C). These findings indicate a strong reduction in ECM deposition and HSC activation, thereby liver fibrogenesis, as a result of STAT3 inhibition in vivo.

### 3.6 STAT3 signaling pathway inhibition ameliorates liver inflammation in acute liver injury mouse model

**Figure 6:**
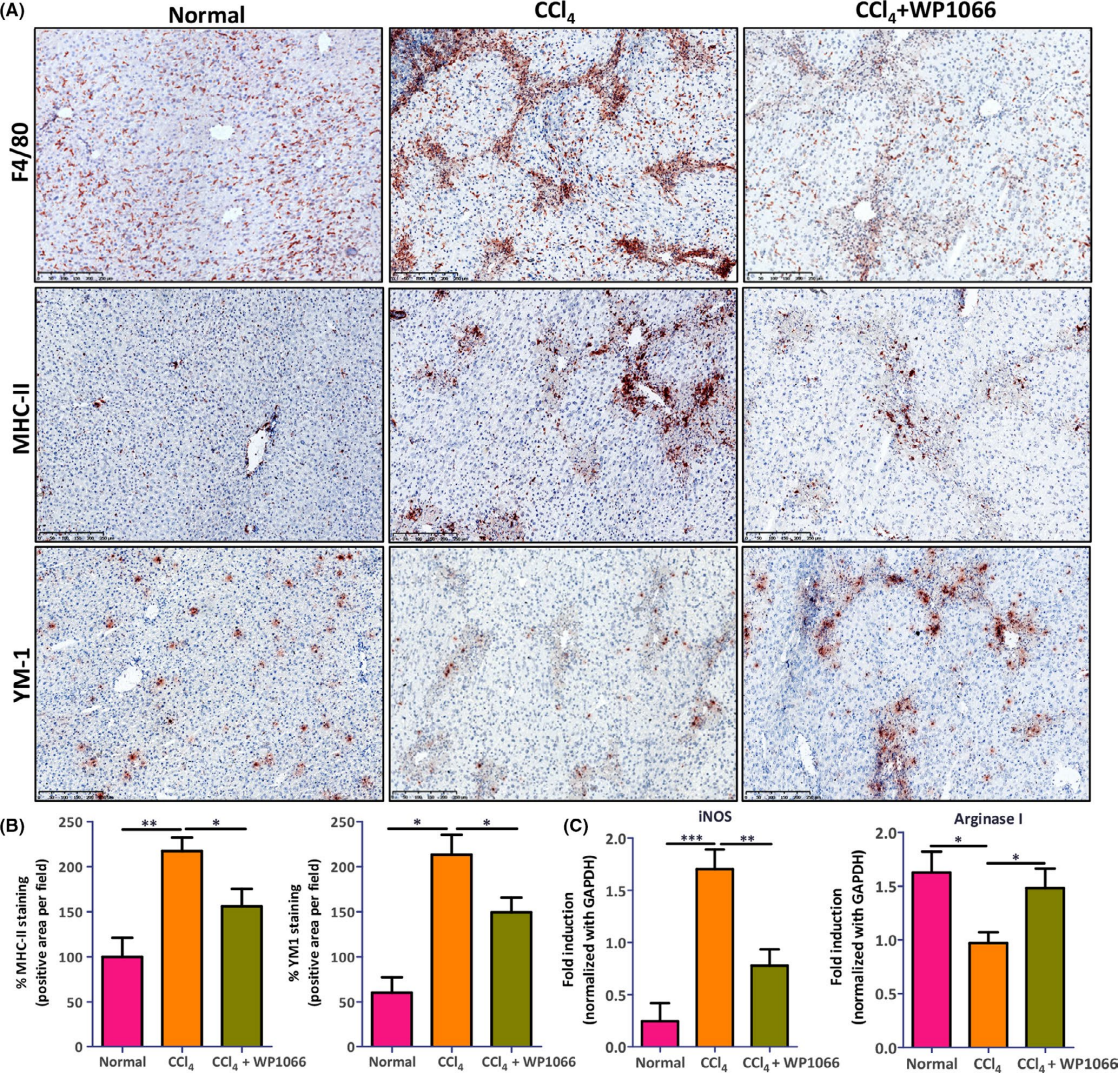
WP1066 ameliorates intrahepatic inflammation in acute CCl_4_‐induced liver fibrogenesis mouse model. (A) Representative microscopic images (n = 5 per group) of stained liver sections and (B) quantitative analysis from normal and CCl_4_ mice with and without WP1066 treatment for F4/80 (macrophage infiltration marker), MHC‐II (M1 marker), and YM‐1 (M2 marker). Data are presented as the mean ± SEM (n = 5 per group). *P < .05, **P < .01 denotes significance. (C) Relative gene expression for iNOS and Arginase I in normal and CCl_4_ mice with and without WP1066 treatment. Data are presented as the mean ± SEM (n = 5 per group). *P < .05, **P < .01, ***P < .001 denotes significance

Acute CCl_4_ liver injury model is a model of acute liver inflammation with increased infiltration and activation of inflammatory M1 macrophages and polarization to M2 restorative macrophages ensues resolution of liver damage. In this study, we also investigated the effects of STAT3 inhibitor WP1066 on macrophage infiltration and polarization in vivo. Significant intrahepatic infiltration and/or de novo intrahepatic proliferation of macrophages was observed following CCl_4_‐induced acute liver injury as confirmed by F4/80 (EMR1, EGF‐like module‐containing mucin‐like hormone receptor‐like 1) immunostainings (Figure 6A). Moreover, we found increased macrophage activation as observed by MHC‐II (major histocompatibility complex class II) staining, and reduced M2 restorative macrophages as shown by YM1 staining's following CCl_4_ treatment as compared to livers from olive oil treated animals (Figure 6A,B). This was further confirmed by mRNA expression levels of M1 marker iNOS and M2 marker Arginase I (Figure 6C). Strikingly, WP1066 inhibited macrophage infiltration, reduced macrophage activation, and increased M2 restorative macrophages (Figure 6A‐C). This effectively shifts the M1/M2 balance towards M2 which alleviates inflammation thus mitigating acute liver injury.

### 3.7 WP1066 inhibition of fibrosis and polarization toward M2 in LPS‐induced human 3D spheroid model

**Figure 7:**
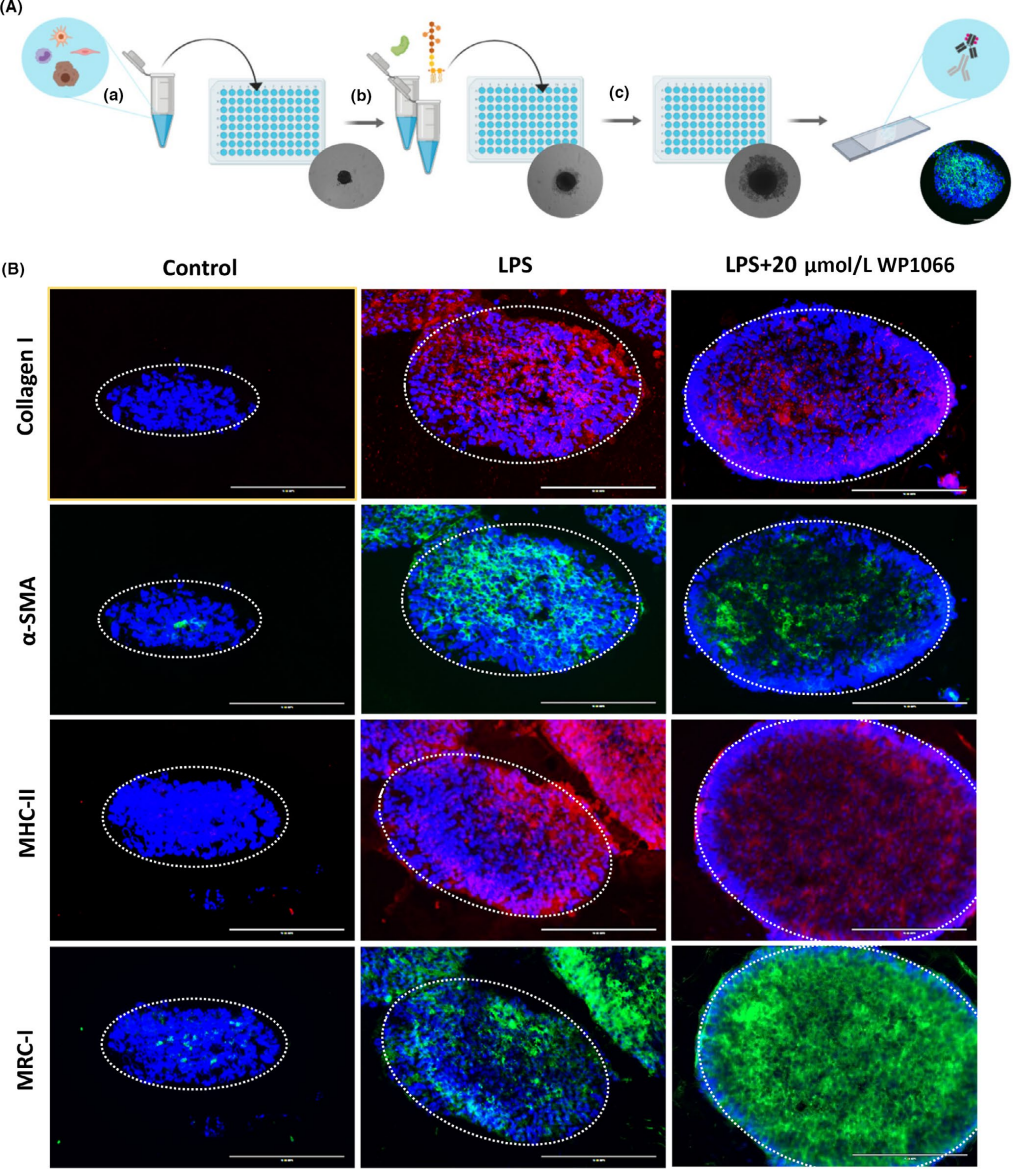
Therapeutic efficacy of WP1066 in human 3D spheroid model. A. Spheroid assay schematic illustration: (a) The cell mixture containing HepG2, LX2, HUVECS, and THP1 was plated in 96‐well ULA plates at a density of 1,200 cells/uL and grown for 7 d; (b) At day 7, spheroids were treated with or without LPS and WP1066; (c) After 7 d of treatment, the spheroids were retrieved, snap frozen, and sectioned. The corresponding cryosections were used for immunostaining. B. First panel of images depicts 3D spheroids stained with fibrosis marker, collagen I (in red); second panel depicts 3D spheroids stained with HSC activation marker, α‐SMA (in green); third panel of images presents 3D spheroids stained with macrophage activation marker, MHC‐II (in red); and fourth panel of images depicts 3D spheroids stained with M2 macrophage marker, mannose receptor (in green). Blue color shows DAPI nuclear staining. The data presented here represent n = 5 spheroids

We further tested WP1066 in a developed human 3D spheroid model (Figure 7A) whereby 3D‐spheroids were formed from human hepatocytes, human hepatic stellate cells, human monocytes, and human endothelial cells as described elsewhere.^15^ We treated spheroids with LPS to mimic acute liver damage, and thereafter incubated with WP1066 to investigate the effects of STAT3 inhibition on LPS‐induced HSCs‐specific and macrophage‐specific parameters. We found that treatment with LPS increased HSCs activation as depicted by collagen‐I, α‐SMA staining, as well as proinflammatory macrophage activation as shown by increased MHC‐II (major histocompatibility complex class II) expression, while reducing M2 restorative macrophage‐specific marker MRC1 (mannose receptor C‐type 1) (Figure 7B). Significantly, treatment with WP1066 dose dependently inhibited collagen I, α‐SMA, as well as MHC‐II expression while increasing MRC1 expression (Figure 7B).

## 4 DISCUSSION

In this study, we have investigated the therapeutic efficacy of selective STAT3 antagonist WP1066 in fibroblasts, inflammatory macrophages, in acute CCl_4_‐induced liver injury murine model in vivo, and LPS‐induced‐3D human spheroid model. We have demonstrated that STAT3 is overexpressed in fibrotic human livers, in activated hepatic stellate cells, and inflammatory macrophages. Moreover, we have shown that WP1066 inhibits HSCs activation and inflammatory macrophages in vitro and in vivo in acute liver injury in mice and 3D spheroids model.

HSCs and macrophages are the core components of acute liver injury^24^ and simultaneous inhibition of these two cell types is essential for the alleviation of liver fibrosis. Studies have shown role of STAT3 pathway in tissue fibrosis.^10^, ^11^, ^12^, ^13^, ^14^, ^25^, ^26^ In particular, STAT3 has been shown to be overexpressed in liver cirrhosis,^26^ and is implicated in aberrant fibroblasts activation in fibrogenesis including systemic sclerosis,^10^ as well as macrophage activation in vagus nerves.^25^ Based on our findings, STAT3 expression is overexpressed and STAT3 signaling pathway is activated in HSCs and inflammatory macrophages, and hence involved in the pathogenesis of acute liver damage. Our findings also corroborate with the previous studies (Chakraborty et al, 2017) documenting that inhibition of STAT3 pathway reduces the activation and contractility of hepatic myofibroblasts.^10^

Importantly, the role of STAT3 pathway in inflammation and inflammatory macrophages remained unknown. Our results further demonstrate the overexpression of STAT3 in LPS/IFNγ‐stimulated pro‐inflammatory M1 macrophages as compared to control and IL‐4/IL‐13 stimulated restorative M2 macrophages. We have shown that STAT3 inhibition reduces inflammatory markers both in vitro and in vivo. Furthermore, we establish that STAT3 inhibition reduces intrahepatic inflammation by reducing the accumulation of M1 inflammatory macrophages while promoting polarization to restorative M2 phenotype macrophages, consecutively favoring regression of liver injury.

The therapeutic effects of STAT3 inhibition in HSCs could be mediated by the combined action of JAK, SRC, c‐ABL, and JNK kinases as suggested earlier.^10^ Since STAT3 pathway activation was induced by TGFβ in HSCs this suggests that STAT3 might also be involved in the TGFβ‐induced signaling pathways that is, Smad2, Smad3, Akt, and ERK as recently suggested by Wang et al.^27^

In conclusion, we have shown the implication of the STAT3 signaling pathway in HSCs, inflammatory macrophages, and acute liver injury. Moreover, we have demonstrated that suppression of STAT3 signaling pathway using WP1066 significantly reduced HSC activation, inflammatory macrophages in vitro, in acute liver injury mouse model, as well as in human 3D spheroid model. To our best knowledge, this is the first comprehensive study describing the role of STAT3 in HSCs and macrophages and demonstrating amelioration of acute liver injury attributed to the inhibition of HSCs and macrophages. Considering its potential effects on inflammatory macrophages and HSCs, STAT3 pathway is also a promising therapeutic target that can be explored in non‐alcoholic fatty liver disease (NAFLD), one of the most common liver‐related complications worldwide. This study suggests that selective inhibition of STAT3 pathway using WP1066 might be a promising therapeutic strategy to inhibit inflammatory and fibrotic diseases.

## CONFLICT OF INTEREST

The authors declare no competing financial interests.

## AUTHOR CONTRIBUTIONS

RB designed research. BOA, AVG, AO‐P, and RB performed the research and analyzed the data. AVG and RB wrote the paper. RB received grants and provided resources for conducting this study. RB revised and edited the manuscript.
