# Supplementary material for: Pharmacological inhibition of STAT3 pathway ameliorates acute liver injury in vivo via inactivation of inflammatory macrophages and hepatic stellate cells
Source: FASEB Bioadv. 2020 Jan 3;2(2):77–89. doi: 10.1096/fba.2019-00070 (PMC7003653; doi:10.1096/fba.2019-00070)
Supplement: Supplementary file 1 [file FBA2-2-77-s001.pdf]

Pharmacological inhibition of STAT3 pathway ameliorates acute liver injury in vivo via inactivation of inflammatory macrophages and hepatic stellate cells

Running Title: STAT3 inhibition ameliorates acute liver injury

AUTHORSHIP:

Büsra Öztürk Akcora[1], Alexandros Vassilios Gabriël[1], Ana Ortiz-Perez[1], Ruchi Bansal[1,2*]

[1]Department of Biomaterials Science and Technology, Technical Medical Centre, Faculty of Science and Technology, University of Twente, Enschede, The Netherlands.

[2]Department of Pharmacokinetics, Toxicology and Targeting, Groningen Research Institute of Pharmacy, University of Groningen, The Netherlands.

***Corresponding Author**

Dr. Ruchi Bansal, PhD

Assistant Professor

Department of Biomaterials, Science and Technology

Technical Medical Centre, Faculty of Science and Technology,

University of Twente, Drienerlolaan 5, 7522 NB, Enschede The Netherlands

Phone: +315-34893115

Email: R.Bansal@utwente.nl

Supplementary Table 1: Antibodies used for the immunohistochemistry

Primary Antibody	Source	Dilution
Monoclonal Rabbit anti-α-SMA	Cell signaling	1:100
Polyclonal goat anti-collagen I	Southern Biotech	1:100
Polyclonal goat anti-collagen III	Southern Biotech	1:100
Polyclonal goat anti-desmin	Santa Cruz	1:100
Polyclonal goat anti-vimentin	Santa Cruz	1:100
Monoclonal rat anti-MHC Class II	Santa Cruz	1:100
Polyclonal goat anti-YM-1 (Biotinylated Anti-mouse chitinase-3-like 3/ECF-L)	R and D systems	1:100
Monoclonal rabbit Mannose receptor	Abcam	1:100
Monoclonal rat anti-F4/80	Bio-Rad	1:100

Secondary Antibody	Source	
Polyclonal goat anti-rabbit IgG	DAKO	1:100
Polyclonal rabbit anti-mouse IgG	DAKO	1:100
Polyclonal goat anti-mouse IgG	DAKO	1:100
Polyclonal rabbit anti-goat IgG	DAKO	1:100
Polyclonal goat anti-rat IgG	Southern Biotech	1:100
Donkey anti-goat Alexa-594	Life Technologies	1:200
Donkey anti-rabbit Alexa-488	Life Technologies	1:200

Supplementary Table 2: Antibodies used for the western blot

Primary Antibody	Source	Dilution
Polyclonal rabbit anti-pSTAT3	Cell signaling	1:500
Monoclonal mouse anti-β-actin	Sigma	1:2000
**Secondary Antibody**	**Source**	
Polyclonal goat anti-rabbit IgG	DAKO	1:1000
Polyclonal rabbit anti-goat IgG	DAKO	1:1000
Polyclonal goat anti-mouse IgG	DAKO	1:1000

Supplementary Table 3: Sequence of the mouse primers used for quantitative real-time PCR

Gene	Forward primer sequence (5’-	Reverse primer sequence (5’-3’)	Accession no.
Collagen 1α1	TGACTGGAAGAGCGGAGAGT	ATCCATCGGTCATGCTCTCT	NM_007742.3
Desmin	ATGCAGCCACTCTAGCTCGT	CTCATACTGAGCCCGGATGT	NM_010043.1
ACTA2 (α-SMA)	ACTACTGCCGAGCGTGAGAT	CCAATGAAAGATGGCTGGAA	NM_007392.2
GAPDH	ACAGTCCATGCCATCACTGC	GATCCACGACGGACACATTG	NM_008084.2
STAT3	CCAACGACCTGCAGCAATAC	GCTCAGCACCTTCACCGTTA	NM_213659.3
iNOS or NOS2	GGTGAAGGGACTGAGCTGTT	GCTACTCCGTGGAGTGAACAA	NM_010927.4
IL-6	TGATGCTGGTGACAACCACGGC	TAAGCCTCCGACTTGTGAAGTGTA	NM_031168.1
IL-1β	GCCAAGACAGGTCGCTCAGGG	CCCCCACACGTTGACAGCTAGG	NM_008361.3
CCL2	GTGCTGACCCCAAGAAGGAA	GTGCTGAAGACCTTAGGGCA	NM_011333.3
CCR2	AGGAGCCATACCTGTAAATGC	TGTGGTGAATCCAATGCCCT	NM_009915.2
ARG1	GTGAAGAACCCACGGTCTGT	CTGGTTGTCAGGGGAGTGTT	NM_007482.3

Supplementary Table 4: Sequence of the human primers used for quantitative real-time PCR

Gene	Forward primer sequence (5’-3’)	Reverse primer sequence	Accession no.
Collagen 1α1	GTACTGGATTGACCCCAACC	CGCCATACTCGAACTGGAAT	NM_000088.3
ACTA2 (α-SMA)	CCCCATCTATGAGGGCTATG	CAGTGGCCATCTCATTTTCA	NM_001613.2
Vimentin	AAATGGCTCGTCACCTTCGT	CAGCTTCCTGTAGGTGGCAA	NM_003380.4
GAPDH	TCCAAAATCAAGTGGGGCGA	TGATGACCCTTTTGGCTCCC	NM_001256799

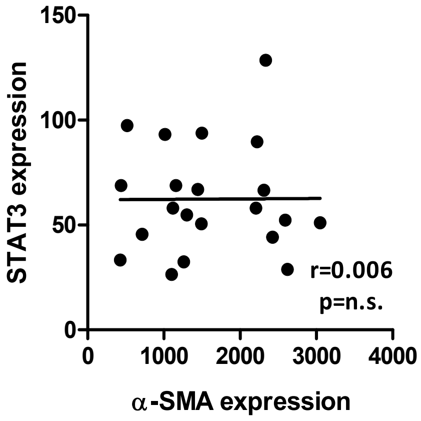

Fig. S1: STAT3-αSMA correlation analysis in cirrhosis patient livers. Correlative analysis of STAT3 and α-SMA expression of cirrhotic liver tissues versus healthy controls. Data are presented as mean ± SEM; control (n=8) and cirrhosis (n=13). *p<0.05, **p<0.01 denotes significance. r denotes spearman's coefficient and p denotes two-tailed P value calculated using non-parametric spearman's correlative analysis with Gaussian approximation.

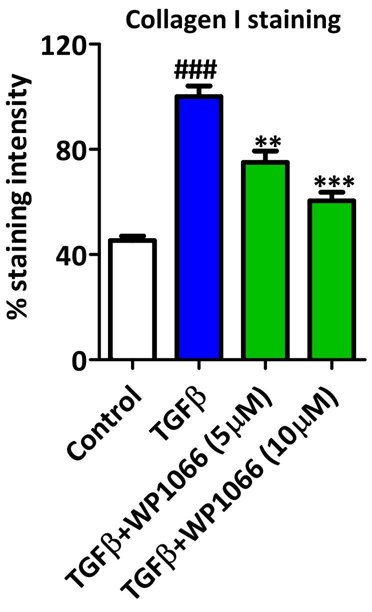

Fig. S2: Inhibitory effect of WP1066 on TGFβ activated mouse 3T3 fibroblasts. The graph showing % collagen-I staining intensity evaluated in control and TGFβ-activated 3T3 fibroblasts with and without treatment with different concentrations (5.0 and 10.0μM) for 24hrs as assessed using ImageJ quantitative analysis of images (in Figure 2B). The data was normalized with TGFβ-activated 3T3 fibroblasts (at 100%). Data are presented as the mean ± SEM (n = 5). ###p<0.001 denotes significance versus control while **p<0.01 and ***p<0.001 denotes significance versus TGFβ-activated fibroblasts.

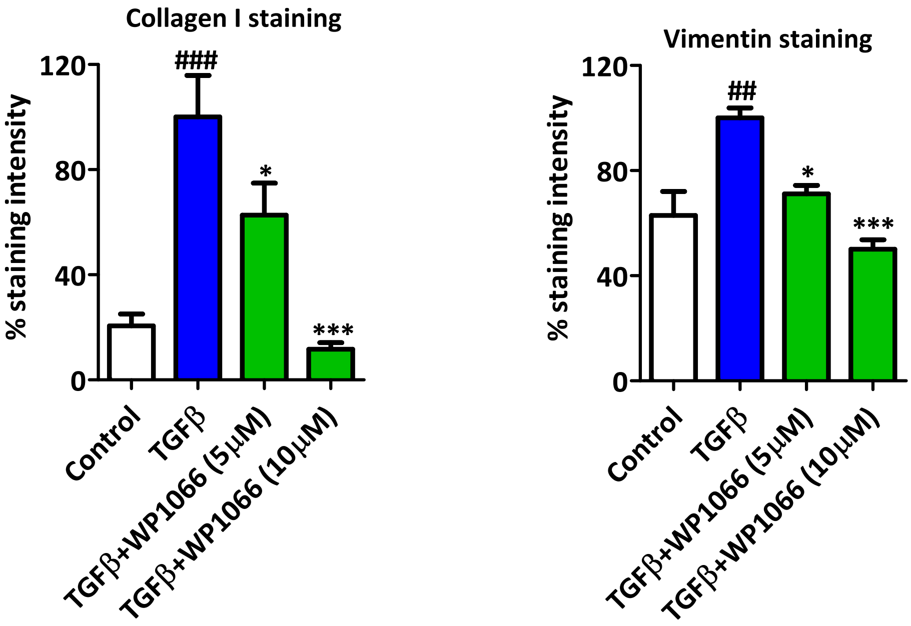

Fig. S3: Inhibitory effect of WP1066 on TGFβ activated human HSCs (LX2 cells). The graph showing % collagen-I and vimentin staining intensity evaluated in control HSCs and TGFβ-activated HSCs with and without treatment with different concentrations (5.0 and 10.0μM) for 24hrs as assessed using ImageJ quantitative analysis of images (in Figure 2B). The data was normalized with TGFβ-activated HSCs (at 100%). Data are presented as the mean ± SEM (n = 5). ###p<0.001 denotes significance versus control while **p<0.01 and ***p<0.001 denotes significance versus TGFβ-activated HSCs.

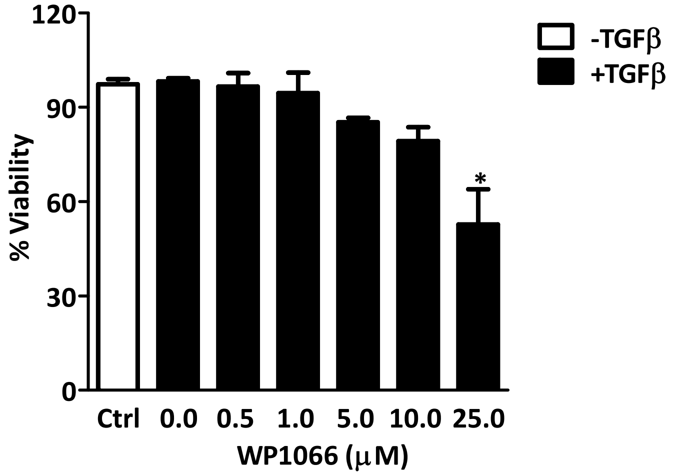

Fig. S4: % Cell viability in human HSCs (LX2 cells). The graph showing % cell viability of control HSCs and TGFβ-activated HSCs with and without treatment with different concentrations (0, 0.5, 1.0, 5.0, 10.0 and 25.0μM) for 24hrs as assessed using Alamar blue assay. The data was normalized with TGFβ-activated HSCs. Data are presented as the mean ± SEM (n = 5). ##p<0.01 denotes significance versus control while *p<0.05, **p<0.01 and ***p<0.001 denotes significance versus TGFβ-activated HSCs.

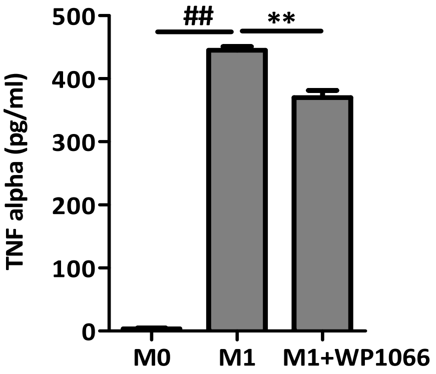

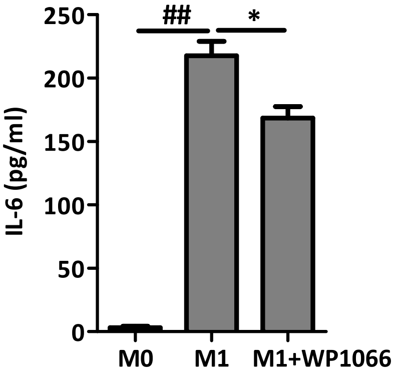

Fig. S5: WP1066 inhibits cytokine release from inflammatory M1 macrophages: Protein expression/release of TNF alpha and IL-6 as assessed in culture supernatant from M0, M1 and M1 treated with 10µM WP1066. Data are presented as the mean ± SEM (n = 5). ##p<0.01 denotes significance versus M0 macrophages while *p<0.05 and **p<0.01 denotes significance versus M1 macrophages.

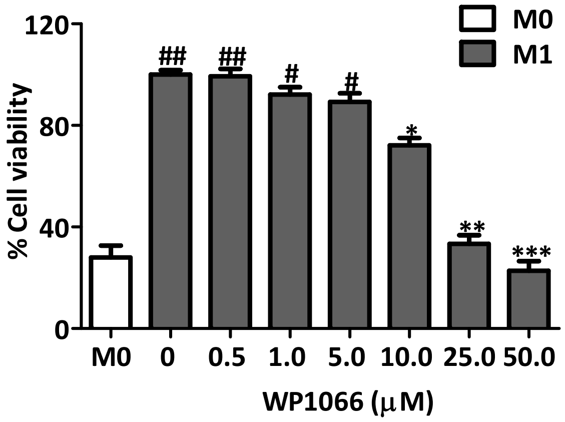

Fig. S6: % Cell viability in murine RAW macrophages. The graph showing % cell viability of control M0 macrophages and M1 macrophages with and without treatment with different concentrations (0, 0.5, 1.0, 5.0, 10.0, 25.0 and 50.0µM) for 24hrs as assessed using Alamar blue assay. The data was normalized with M1 macrophages. Data are presented as the mean ± SEM (n = 5). ##p<0.01 denotes significance versus M0 macrophages while *p<0.05, **p<0.01 and ***p<0.001 denotes significance versus M1 macrophages.
